# Synthesis and characterization of nano-micronutrient fertilizer and its effect on nutrient availability and maize (*Zea Mays* L.) productivity in calcareous soils

**DOI:** 10.1038/s41598-025-11273-7

**Published:** 2025-07-16

**Authors:** Dalal H. Sary, Mahmoud E. Abd El-Aziz

**Affiliations:** 1 Soil, Water and Environment Research Institute, Agricultural Research Center, 9 Cairo Univ. St, P.O. 12112, Giza, Egypt; 2 Polymers & Pigments Department, National Research Centre, 33 El Bohouth St., Dokki, P.O. 12622, Giza, Egypt

**Keywords:** *Zea mays* L., Nano micronutrient, Calcareous, Soil, Plant sciences, Nanoscience and technology

## Abstract

Agriculture in calcareous soil faces challenges such as high calcium carbonate content, low organic matter, and poor availability of nutrients. Therefore, a field experiment was done during the summer seasons of 2022 and 2023 at the Nubaria Research Station in Egypt to study the effect of nano-fertilizers (NFs) on maize productivity and nutrient availability. The field experiment was done through a randomized completely block design with treatments: Control, Nano-Zn 20 mg/L, Nano-Zn 40 mg/L, Zn-chelate 2 g/L, Nano-Mn 20 mg/L, Nano-Mn 40 mg/L, Mn-chelate 2 g/L, Nano-Mo 20 mg/L, Nano-Mo 40 mg/L and ammonium molybdate (AMo) 250 mg/L. The synthesized nano-micronutrient fertilizers were analyzed by X-ray diffraction (XRD) and transmission electron microscopy (TEM). The results showed that 40 mg/L of NFs was the most effective treatment for most studied traits. The highest maize grain weight per plant (239.4 g), 100-grain weight (40.7 g), and yield (15.1 ton/ha) were obtained with 40 mg/L of Nano-Mo, Zn, and Mn, respectively. The maximum leaves nutrient contents of P (0.98%), K (1.0%), Fe (268 mg/kg), Zn (79 mg/kg), and Cu (24.3 mg/kg) were found in plants treated with Nano-Zn 40 mg/L. While, the highest concentrations of Mn (271.7 mg/kg) and N (3.96%) on the leaves were obtained with 40 mg/L of Nano-Mn and Mo, respectively. When compared to traditional fertilizers, NFs showed better plant growth traits, productivity, and nutrient levels.

## Introduction

In Egypt, the area planted with maize is about 0.66 million hectares, producing approximately 5.85 million tons of grain. These grains are used in human food, animal feed, and industrial purposes^1^. Plant growth and quality, as well as soil quality, can be improved by managing the availability of nutrients in the rhizosphere. This is particularly important because the use of synthetic chemical fertilizers, which can be costly for farmers and harmful to the environment, has made micronutrient management a global concern^2^. Recently, crop productivity and yield can be enhanced by improving nutrient efficiency through nanotechnology (NT). It has been employed to produce high-quality nutrient delivery, biological support, and financial stability that boost crop productivity and reduce the high costs associated with traditional fertilizers^3,4^. Also, it provides an eco-friendly alternative to traditional fertilizers by improving physical, chemical, and biological properties^5,6^.

Nano-fertilizers (NFs), ranging in size from 1 to 100 nm, are characterized as a higher alternative to traditional fertilizers due to their higher nutrient use efficiency and enhanced plant tolerance to biotic and abiotic stresses^7,8^. Also, NFs are known as a “smart system of nutrients”^9,10^. NFs offer a smart delivery system, stimulating nutrient utilization efficiency through targeted, controlled, or slow-release mechanisms^11,12^. These fertilizers optimize plant nutrition and offer a cost-effective alternative to traditional fertilizers in addition to improving the growth and production of plants and enhancing the micronutrient efficiency in soils^13,14^. So, NFs support sustainable global food production and provide a new method for enhancing nutrient supply^15,16^. NFs are distinct in that they have higher mobility, a larger surface area-to-volume ratio, longer-lasting effects, and easier penetration into plant tissues. This improves nutrient absorption and availability, ultimately increasing crop yield and quality^17^. Moreover, NFs enhance microbial count, nutritional content, bioavailability, and overall soil health^18^.

Understanding the interactions between crops and nanoparticles (NPs) can help us better understand nutrient absorption, mobilization, and accumulation^19^. NPs can enter plant tissues through many pathways, such as soil microbes and root exudates in roots, or through stomata, plasmodesmata, and permeable regions in leaves. Despite limitations due to cell wall pore size and the Casparian strip, nanoparticles can be transported between cells via endocytosis, carrier interactions, or plasmodesmata^20^. The foliar application, through which nutrients are absorbed via nanopores in leaf plasmodesmata, is one of the most efficient techniques for nutrient absorption^21^.

Zinc (Zn) nutrient fertilizer is essential for enzyme catalytic activity, but its effectiveness can be inhibited by adsorption by soil clay complexes. Zn-NFs work to improve transport within plants and reduce fixation in the soil compared to traditional sources of Zn^22^. Research shows that foliar application of 600 mg/L of ZnO-NPs on maize can significantly improve plant growth, yield, and grain quality in addition to overcoming issues like rapid immobilization in calcareous soils^17^. The application of Zn as ZnO-NPs may also reduce risks like leaf burn, which can occur with soluble Zn forms^23^. Manganese (Mn) is important for photosynthesis, respiration, and nitrogen metabolism in plants. Mn-NFs may influence photochemical processes in soil-plant systems^24^. Mn-NFs, in particular, have shown a potential to safely increase crop productivity^25^. Mn-NFs are more effective micronutrient sources than commercially available Mn fertilizer^26^. The employment of Mn-NPs as a foliar fertilizer facilitated their passage through plant cell pores more efficiently and improved manganese nutrition and plant performance^23^. Molybdenum (Mo) is another crucial micronutrient, playing an important role in the nitrate reductase enzyme, which is necessary for nitrogen assimilation^27^. Mo-NPs have been found to significantly improve the microbial characteristics of the rhizosphere in agriculturally beneficial microbes^28^. Also, they promote plant growth through complex physiological processes, including enhanced enzyme activity and nitrogen uptake. They support nutrient absorption and homeostasis by increasing root volume, surface area, and absorption efficiency^29^.

This study aims to investigate the application of nano-fertilizers as an innovative technology to maximize maize productivity under calcareous soil conditions.

## Materials and methods

### Preparation of nano-nutrient fertilizer

The applied materials, such as manganese nitrate, ammonium molybdate (AMo), citric acid, and zinc acetate, were purchased from Sigma-Aldrich Company. Sodium hydroxide and ammonium hydroxide were purchased from S.D. Fine-Chem.

ZnO-NPs were made by refluxing zinc acetate (3.942 g) in 1 L of ethanol containing 1.44 g of NaOH for two hours at 70 °C. After obtaining and purifying ZnO-NPs using DI-water, they were centrifuged for 10 min at 5000 rpm to produce a fine white powder that was dried for 24 h at 60 °C^30^.

After dissolving 10 g of manganese nitrate (Mn (NO_3_)_2_•4H_2_O) in 5 mL of water at 80 °C while stirring for 10 min, the concentrated solution was baked at 100 °C for 24 h to convert into a black viscous liquid. The deionized water was then added to a viscous liquid and centrifuged three times for 15 min at 10,000 rpm before the black particles were dried at 100 °C to get finally MnO_2_-NPs.^31^.

The sol-gel method was used to create MoO_3_-NPs. Ammonium hydroxide was employed to bring the pH down to 7 after AMo (11.6 g) and citric acid (3.8 g) were dissolved in distilled water while being stirred. The resultant solution was heated to 250 °C for an hour to create a powder, and then it was raised to 500 °C for 120 min to create MoO_3_-NPs.^32^.

### Characterization

Transmission electron microscope (TEM; JEM-1230, Japan) set to 120 kV, 600 × 10^3^ magnification, and 0.2 nm resolution, was used to evaluate the shape and size of produced NPs. The NPs’ X-ray diffraction (XRD) patterns were obtained using a Philips X-ray diffractometer (PW 1820 goniometer, PW 1930 generator, and radiation source CuK) and a Diano X-ray diffractometer with radiation source CoKα running at 45 kV.

### Field experiment

A field experiment was done at the El-Nubaria Research Station, Behaira Governorate, Agric. Res. Center, Ministry of Agriculture and Land Reclamation, Egypt, to evaluate the effects of nano micronutrient fertilizers on the growth, yield, and nutritional value of maize plant cultivar under calcareous soil conditions during the summer seasons of 2022 and 2023. The farm’s geographical location is 30° 90´ N, 29° 96´ E, and it is 25 m above sea level.

### Soil properties

The physical and chemical properties of soil are shown in Table 1. Soil physical properties, including the percentage of sand, silt, and clay as well as soil texture class, were studied according to Cottenie et al.^33^. The soil chemical properties, including electrical conductivity (EC), soil reaction (pH), organic matter percentage (OM%), and total calcium carbonate contents, were described by Page, et al.^34^. K_2_SO_4_ (1%), NaHCO_3_ (0.5 N), and NH_4_OAc (1 N; pH 7.0) were used to extract the soil’s available N, P, and K^35,36^. The nitrogen content was measured using the digest’s micro-Kjeldahl distillation method^37^. The ascorbic acid method was used to calorimetrically measure the soil phosphorus content^38^. A flame photometer was used to measure the K concentration^35^. Lindsay and Norvell^39^ used a DTPA solution to determine the available levels of Fe, Mn, Zn, and Mo.

**Table 1:** Physical and chemical properties of the experimental soil (the data are the average for two seasons).

Soil physical properties										
Texture			Sand (%)			Silt (%)			Clay (%)	
loamy sand			79			13.8			7.2	

Soil chemical properties										
CaCO_**3**_ (%)	EC (dS/m)	pH	OM (%)	Nutrients (mg/kg)						
				N	P	K	Fe	Mn	Zn	Mo
28.4	1.4	8.3	0.8	40	3	100	4	2.8	1.04	0.02

### Experimental design

The field experiment was done using a randomized complete block design (RCBD) on 30 plots with three replications and ten treatments. Each plot consists of 4 lines of plantation, 3.5 m long rows, 75 cm row spacing, and 20 cm plant-to-plant spacing in a 10.5 m^2^ plot area. The treatments included; control, Nano-Zn 20 mg/L, Nano-Zn 40 mg/L, Zn-chelate 2 g/L, Nano-Mn 20 mg/L, Nano-Mn 40 mg/L, Mn-chelate 2 g/L, Nano-Mo 20 mg/L, Nano-Mo 40 mg/L and AMo 250 mg/L. These treatments were applied using foliar sprays three times during the growing season.

### Maize cultivar

The maize (single hybrid 166) was obtained from the Agricultural Research Center’s Corn Research Department, Giza, Egypt. The Egyptian Ministry of Agriculture and Land Reclamation recommended adding NPK fertilizer in the following forms: ammonium sulfate (20.5% N), superphosphate (15.5% P_2_O_5_), and potassium sulfate (48% K_2_O). All agricultural operations, such as fertilization, irrigation, weed control, disease control, and others, were done according to the recommendations of the Egyptian Ministry of Agriculture.

### **Vegetative growth and yield components**

Plant height (cm), fresh weight of plant (kg), ear length (cm)/plant, ear diameter (cm), number of rows/ears, number of ears/plants, weight of 100 grains (g), weight of ears (g/plant), and weight of grains (g/plant) as a mean value for two seasons were determined by taking three plant samples from each plot. Grain yield (ton/ha) was calculated by removing and cleaning grains obtained from 1 m^2^ at the center of each plot.

### Biochemical analysis

#### Nutrients in leaves and grains

The harvest plant samples (leaves and grains) were used for nutrient determination. The nitrogen was estimated using the Micro-Kjeldahl method. Then the protein content was calculated using a factor of 6.25. Phosphorus was determined calorimetrically, and potassium content was measured using a flame Photometer. The content of iron, zinc, manganese, and copper was measured by Perkin Elmer Atomic Absorption Spectrophotometer^40^.

#### Nutrients in soil

After harvesting, soil samples were collected, air-dried, ground, and sieved to analyze macro-nutrients (NPK) and micro-nutrients (Fe, Zn, Mn, and Mo) according to methods described in Sect. Soil properties.

### Statistical analysis

The data were analyzed statistically using the least significant differences (LSD) in the means of the data of the two seasons were analyzed statistically^41^.

## Results

### Characteristics of nano-fertilizers

**Fig. 1:**
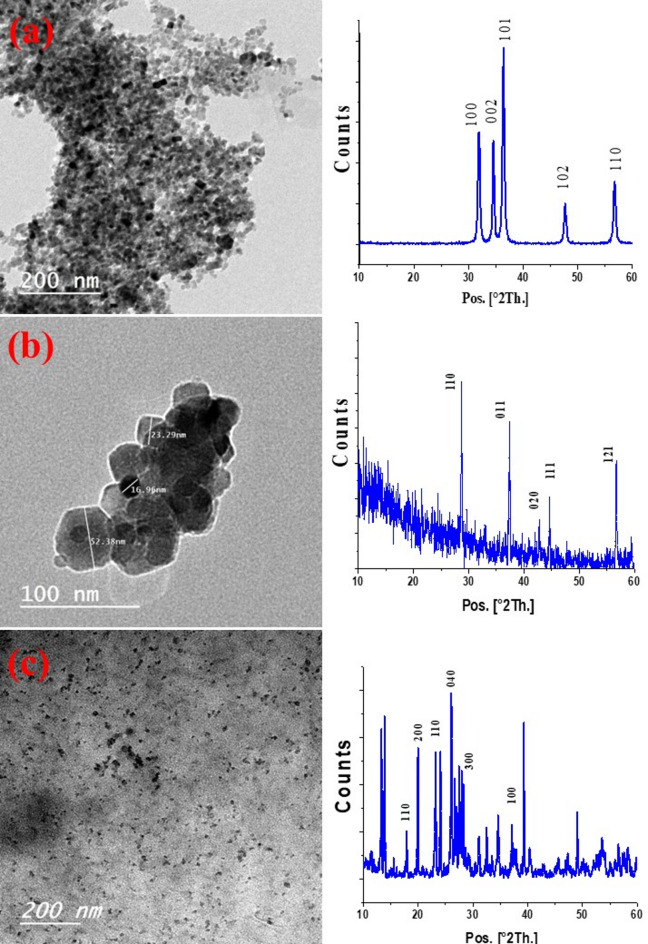
TEM image of (**a**) ZnO-NPs, (**b**) MnO_2_-NPs, and (**c**) MoO_3_-NPs and their corresponding XRD.

Figures 1a, b, **and c** show the morphological structures of ZnO-NPs, MnO_2_-NPs, and MoO_3_ -NPs, respectively, and their corresponding XRD. The as-prepared NPs have particle sizes smaller than 100 nm which are demonstrated by the results. MnO_2_-NPs had a consistent diamond shape and an average particle size of 75 nm, while ZnO-NPs’ TEM picture revealed an average particle size of 25 nm. While the average particle size of MoO_3_-NPs displayed 10 nm.

The crystal structures of the ZnO, MnO_2_, and MoO_3_ -NPs were investigated using X-ray diffraction measurements (Fig. 1). The plans (100), (002), (101), (102), and (110) are associated with the principal ZnO-NPs peaks that were measured at 2θ = 32, 34.4, 36.4, 47.7°, and 56.7°, respectively^42,43^. In addition, The MnO_2_-NPS show three phases known as α-MnO_2_, γ-MnO_2_ and β-MnO_2_ which appeared peaks at 2θ (28.7 and 37.3°), (24.8 and 65°), and (42.82, 46.02, 56.65, and 59.3°), respectively^44,45^. The orthorhombic crystal structure of MoO_3_–NPs is shown by the peaks at 2θ = 13.9, 23.2, 26.1, 34.5, 39.3, and 49° in the MoO_3_–NP pattern^46,47^. The produced nanoparticles’ XRD patterns show that the as-prepared NPs are pure.

### **Vegetative growth and yield components**

**Fig. 2:**
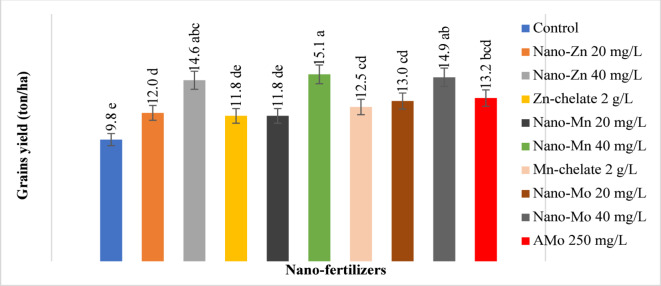
Effects of NFs on yield of maize plant cultivar under calcareous soil.

Table 2 shows that, under calcareous soil conditions, the application of NFs considerably increased the height of maize plants in comparison to the control treatment. The Nano-Mo 40 mg/L treatment produced the highest fresh weight per plant (1.377 Kg). The highest 100-grain weight (40.7 g) was obtained with 40 mg/L Nano-Zn. The Nano-Mo 40 mg/L treatment had a much greater grain weight per plant (239.4 g) and 40 mg/L of Nano-Zn treatment came in second (236.2 g). The Nano-Mn 40 mg/L treatment had the maximum productivity in terms of grain production per hectare, followed by the Nano-Mo 40 mg/L and Nano-Zn 40 mg/L treatments. The treatment 40 mg/L Nano-Mn produced the most grain (15.1 tons/ha) in comparison to the control (9.84 tons/ha), as shown in Fig. 2.

**Table 2:** Effects of NFs on the growth and yield component of maize plant cultivar under calcareous soil conditions (combined analysis of two successive seasons).

Treatments	Plant Height (cm)	Fresh weight (kg) / plant	Weight of 100 grains (g)	Grain weight (g)/plant
**Control**	167b	0.627d	36de	167.4de
**Nano-Zn 20 mg/L**	221.7a	0.920bc	38.3abcd	191.8abcd
**Nano-Zn 40 mg/L**	228a	0.990b	40.7a	236.2ab
**Zn-chelate 2 g/L**	225.3a	0.930bc	39abc	183.1abc
**Nano-Mn 20 mg/L**	212a	0.776 cd	35.3e	189.8e
**Nano-Mn 40 mg/L**	220.3a	0.964b	36.7cde	209cde
**Mn-chelate 2 g/L**	218.7a	0.888bc	39abc	234.8abc
**Nano-Mo 20 mg/L**	208.3a	0.839bc	38.7abc	207.9abc
**Nano-Mo 40 mg/L**	218a	1.377a	39.7ab	239.4ab
**AMo 250 mg/L**	217a	0.997b	37.3bcde	220.7bcde
**LSD 5%**	23.04	0.169	2.60	33.69

Error bars refer to the LSD_**5%**_ values of the statistical analysis of data.

### Characteristics cob of maize plant

Table 3 shows no significant differences in ear length of maize for each plant with NFs at high concentrations (Nano-Zn 40 mg/L, Nano-Mo 40 mg/L, and Nano-Mn 40 mg/L), but it was significant compared to the control. The Nano-Zn 40 mg/L treatment shows the highest ear diameter value (6.5 cm), number of rows per ear (17.3), and weight of the ear per corn plant (494.7 g) which was significant compared to the other treatments and control. The highest number of ears per corn plant was observed with treatments 40 mg/L Nano-Zn (2.33) and Nano-Mo 40 mg/L (1.88).

**Table 3:** Effects of NFs on characteristics cob of maize plant cultivar under calcareous soil conditions (combined analysis of two successive seasons).

Treatments	Length of ear /plant (cm)	Diameter of ear (cm)	No. rows /ear	No. ears/plant	Weight of ears /plant (g)
**Control**	19.8d	4.4c	11.9c	1.11c	262.3f
**Nano-Zn 20 mg/L**	22 cd	5.2b	14.1bc	1.22c	385.1bcd
**Nano-Zn 40 mg/L**	25.8a	6.5a	17.3a	2.33a	494.7a
**Zn-chelate 2 g/L**	23.3bc	5.7b	15b	1.22c	337.1de
**Nano-Mn 20 mg/L**	22.5c	5.4b	14.8b	1.22c	317.0e
**Nano-Mn 40 mg/L**	23.9abc	5.5b	15.9ab	1.44bc	401.2bc
**Mn-chelate 2 g/L**	23.1bc	5.4b	14.4b	1.44bc	372.5bcd
**Nano-Mo 20 mg/L**	23.5bc	5.3b	14.8b	1.44bc	354.78cde
**Nano-Mo 40 mg/L**	24.9ab	5.6b	14.9b	1.88ab	416.6b
**AMo 250 mg/L**	22.2c	5.6b	14.1bc	1.44bc	358.7cde
**LSD 5%**	2.2	0.68	2.26	0.52	52.1

### Biochemical analysis

#### Nutrient content in leaves

Table 4 illustrates the effects of NFs on the nutrient content in maize leaves grown under calcareous soil conditions. It was noted that the level of nitrogen in the maize plants’ leaves had the highest significance with Nano-Mo 40 mg/L (3.96%) followed by Mn-chelate 2 g/L (3.4%), and Nano-Mn 40 mg/L (3.4%). Phosphorus and potassium content in corn leaves varied significantly between treatments and control. The Nano-Zn 40 mg/L treatment shows the highest significance in phosphorus content (0.98%) and potassium content (1.0%). The Nano-Zn 40 mg/L treatment had a highly significant impact on the iron content in the leaves (268 mg/kg). The content of zinc nutrients in the maize leaves was highly significant with zinc foliar applications, following the order: Nano-Zn 40 mg/L > Nano-Zn 20 mg/L > Zn-chelate 2 g/L. In the same trend, the Mn content in corn leaves was enhanced using foliar treatment of Mn. In addition, the copper content in maize leaves showed a significant difference between both conventional and NFs treatments compared with the control.

**Table 4:** Effect of NFs on leaves nutrients content of maize plant cultivar under calcareous soil conditions (combined analysis of two successive seasons).

Treatments	N	P	K	Fe	Zn	Mn	Cu
	(%)			(mg/kg)			
**Control**	1.44f	0.04f	0.5d	118.3j	25.7 g	153 g	8.6 g
**Nano-Zn 20 mg/L**	3.04 cd	0.27e	0.62 cd	203.3c	58.7b	205.7cde	16.6bc
**Nano-Zn 40 mg/L**	3.88ab	0.98a	1.0a	268a	79a	215c	24.3a
**Zn-chelate 2 g/L**	2.52de	0.66b	0.59d	231.7b	57.3b	211 cd	18.2b
**Nano-Mn 20 mg/L**	3.04 cd	0.39 cd	0.59bc	166.3f	42de	251b	13.3e
**Nano-Mn 40 mg/L**	3.4abc	0.44c	0.84b	186d	49c	271.7a	15.1 cd
**Mn-chelate 2 g/L**	3.4abc	0.29e	0.6 cd	176e	45 cd	238b	14de
**Nano-Mo 20 mg/L**	2.32e	0.41 cd	0.81b	131.7i	31f	174.7f	11.2f
**Nano-Mo 40 mg/L**	3.96a	0.40 cd	0.84b	152.7 g	39.3e	199de	12.7ef
**AMo 250 mg/L**	3.24bc	0.36d	0.73bc	142.3 h	37.3e	195e	11.6f
**LSD 5%**	1.7	0.155	0.127	7.6	5.3	13.5	1.65

N (nitrogen), P (Phosphorus), K (potassium), Fe (iron), Zn (zinc), Mn (manganese), and Cu (copper).

#### Nutrient content in the grains

The data in Table 5 illustrated that treating maize grains with Nano-Mo 40 mg/L showed increasing noticeably for both protein (10.25%) and nitrogen contents in grains (1.64%). Phosphorus content was significantly higher with the Nano-Zn 40 mg/L treatment (0.43%). The grains’ potassium content shows a high content with a foliar application with NFs treatments compared to traditional fertilizer (Mn-chelate 2 g/L and AMo 250 mg/L). Also, it was significant in the treatments with Nano-Mo 40 mg/L, Nano-Zn 40 mg/L, Zn-chelate 2 g/L, and Nano-Zn 20 mg/L compared to the control. Furthermore, Nano-Zn 40 mg/L treatment shows the highest significant iron content of corn grains (125 mg/kg). The zinc content in corn grains showed the highest values with a foliar treatment using Nano-Zn 40 mg/L, Nano-Zn 20 mg/L, and Zn-chelate 2 g/L. In addition, the manganese content in corn grains was significantly higher with the treatments Nano-Mn 40 mg/L, Nano-Mn 20 mg/L, and Mn-chelate 2 g/L, which recorded values of 22.3, 18.8, and 17.9 mg/kg, respectively. Indeed, foliar fertilization with a specific nutrient is responsible for improving the content of that nutrient in the maize grains, which can be seen for both foliar applications of manganese and zinc. Furthermore, the foliar application with high concentrations of Nano-Zn, Mn, and Mo significantly improved the copper content in corn plant grains with values of 9.6, 7.2, and 6.63 mg/kg, respectively.

**Table 5:** Effect of NFs on protein and grains nutrient content of maize plant cultivar under calcareous soil conditions (combined analysis of two successive seasons).

Treatments	Protein	N	P	K	Fe		Zn		Mn		Cu
	(%)				(mg/kg)						
**Control**	5.5 g	0.88 g	0.08e	0.60c	61e		11.7i		7.1 h		4.23e
**Nano-Zn 20 mg/L**	6.925ef	1.108ef	0.30bc	0.74ab	82.3b		24b		10.8f		5.17de
**Nano-Zn 40 mg/L**	9.975ab	1.596ab	0.43a	0.76a	125a		27a		17.1c		7.2b
**Zn-chelate 2 g/L**	6.125 fg	0.98 fg	0.35b	0.75ab	86b		22c		14.1d		5.67 cd
**Nano-Mn 20 mg/L**	9bc	1.44bc	0.23d	0.63c	73c		16.3 g		18.8b		5.9bcd
**Nano-Mn 40 mg/L**	9bc	1.44bc	0.32bc	0.687abc	83.7b		21.3 cd		22.3a		9.6a
**Mn-chelate 2 g/L**	7.75de	1.24de	0.32bc	0.65c	75c		19.3ef		17.9c		6.1bcd
**Nano-Mo 20 mg/L**	8.375dc	1.34dc	0.29 cd	0.62c	63.3de		14 h		9.03 g		4.97de
**Nano-Mo 40 mg/L**	10.25a	1.64a	0.32bc	0.76a	67.3d		20de		14.7d		6.63bc
**AMo 250 mg/L**	7.125ef	1.14ef	0.27 cd	0.66bc	64.3de		17.7 fg		12.47e		5.4cde
**LSD 5%**	1.75	1.01	0.57	0.089	4.68		1.99		0.856		1.32

N (nitrogen), P (Phosphorus), K (potassium), Fe (iron), Zn (zinc), Mn (manganese), and Cu (copper).

#### Nutrient in soil

The results in Table 6 show the effect of various applied treatments on the micronutrient content in the soil. According to the data in Table 6, the foliar application of both nano and traditional micronutrient fertilizers significantly improves the amount of available nitrogen in the soil compared to the control. The results represent that the available phosphorus in calcareous soil showed the highest significance in the treatments Nano-Mo 40 mg/L, Nano-Zn 40 mg/L, and Nano-Mo 20 mg/L with values of 16.2, 15.53, and 15.43 mg/kg, respectively. Also, the soil potassium content showed significant differences between all the treatments compared to the control, where the highest value (289.5 mg/kg) was recorded with the Nano-Mo 40 mg/L treatment. The treatment Nano-Zn 40 mg/L showed the highest available amount of iron in the soil (10.8 mg/kg). While the highest available amount of zinc in the soil was obtained in the order Nano-Zn 40 mg/L (2.83 mg/Kg), Zn-chelate 2 g/L (2.53 mg/Kg), followed by Nano-Zn 20 mg/L (2.16 mg/Kg), respectively.

Additionally, the maximum significance of manganese was found in the order of Nano-Mn 40 mg/l, Nano-Mn 20 mg/l, and Mn-chelate 2 g/l. In the same vein, the treatments Nano-Mo 40 mg/l, Nano-Mo 20 mg/l, and AMo 250 mg/l demonstrated the highest levels of molybdenum in the soil. It seems that foliar fertilization with a specific nutrient is responsible for improving the content of that nutrient in the soil compared to other treatments. This was demonstrated for molybdenum, manganese, and zinc (Table 6).

**Table 6:** Effect of NFs on nutrients in the soil of maize plant cultivar under calcareous soil conditions (combined analysis of two successive seasons).

Treatments	A. *N*	A. *P*	A. K	Fe	Zn	Mn	Mo
	(mg/kg)						
**Control**	62 g	6.73d	118.5f	6.175f	1.07e	3.29 g	0.02f
**Nano-Zn 20 mg/L**	74e	9.35bc	248.2b	6.18f	2.16bc	3.78f	0.08e
**Nano-Zn 40 mg/L**	86c	15.53a	256.5b	10.8a	2.83a	5.71c	0.24bc
**Zn-chelate 2 g/L**	80d	6.9d	190.3d	6.74ef	2.53ab	4.52e	0.21d
**Nano-Mn 20 mg/L**	74e	6.87d	140.6e	8.115de	1.89 cd	7.14a	0.21d
**Nano-Mn 40 mg/L**	100b	10.82b	215.1c	9.08bcd	1.997bcd	7.42a	0.24abc
**Mn-chelate 2 g/L**	66f	8.05 cd	173.7d	8.79 cd	1.957bcd	7.14a	0.217d
**Nano-Mo 20 mg/L**	84c	15.43a	223.4c	10.18abc	2.14bc	5.03d	0.25ab
**Nano-Mo 40 mg/L**	104a	16.2a	289.5a	10.42ab	2.2bc	6.15b	0.27a
**AMo 250 mg/L**	99b	10.63b	264.7b	6.67ef	1.5de	5.29d	0.25ab
**LSD 5%**	2.02	1.82	21.02	1.47	0.6	0.28	0.028

## Discussion

### **Vegetative growth and yield components**

The data demonstrated that the vegetative growth and yield components of corn plants were enhanced at high concentrations of nano-micronutrient fertilizer (40 mg/L). Accordingly, this was reflected in improving the maize grains productivity under calcareous soil conditions. El-Metwally et al.^48^ found that the grain yield was improved when NFs were applied at varying doses. NFs may enhance maize productivity by improving the plant uptake of nutrients and promoting pigment synthesis^49^. The foliar application of ZnO-NPs showed the highest vegetative growth of plants, especially the plant height^50^. Previous studies show nano-micronutrients significantly increase plant height, ear weight, 100-grain weight, and grain yield, with the highest values observed in foliar addition of nano-Fe + Zn + Mn^51^. The foliar application of NFs, namely 10 mg/L zinc NPs, which resulted in enhanced plant height and fresh weight, may be responsible for the increase in maize yield and its constituent parts^52^. Also, the morphological and yield characteristics of maize were greatly enhanced by a nano-urea spray^53^. Plant height, number of ears, grain weight, and yields were affected by the application of NPs; the maximum values were recorded at 100 g nano/fed^54^. The application of ZnO-NPs had a substantial impact on maize yield parameters, such as total grain weight, 1000 grain weight, and yield^55^. Reddy et al.^56^ have shown that applying 50% nitrogen via urea and nano urea, along with nano zinc, greatly boosts grain output and plant height by 8926 kg/ha and 195.80 cm, respectively. The Nano-Zn granules had the highest 100-grain weight^57^. Also, the use of ZnO-NPs enhances the characteristics of corn^58^. Plants treated with 30 mg/L of MnO2-NPs showed improved vegetative growth, which resulted in a significant increase in plant length and yield^14^. Al-Kraiti et al.^59^ claim that NFs greatly improve the maize plant’s vegetative growth.

### Nutrient content in leaves

According to the findings, the most effective treatment for raising the nitrogen content of maize leaves was Nano-Mo 40 mg/L. This is because molybdenum not only contributes to nitrogen metabolism but also enters the composition of the enzymes nitrogenase and nitrate reductase^43,47^. According to Muñoz-Márquez, et al.^3^, nano Mo improves the efficiency and assimilation of nitrogen. Al-Saray and Al-Rubaee^60^ claim that the application of NFs to maize plants raised their nitrogen levels, presumably as a result of the nutrients and accessible nitrogen building up when the plants were treated with NFs. The current investigation showed that the corn leaves had a high content of phosphorus and potassium, with a foliar application at a concentration of 40 mg/L for both Nano-Zn, Mn, and Mo. Khardia et al.^61^ showed that the plots treated with NFs provided better nutrients. The production of maize increased with the foliar application of nano zinc, which greatly enhanced P availability and uptake^62^. Also, the nano-macro and micronutrient treatments had the highest percentages of NPK compared to the control^63^.

According to this study, iron and copper content in the leaves of maize grown in calcareous soil was improved by foliar treatments with NFs containing zinc and manganese. These findings concur with those of Reshma & Meenal^52^, who discovered that foliar application of nano zinc enhanced the amount of zinc in plant biomass, resulting in better yield and nutrient recovery. When compared to the control, nano-Zn and Mn at 1500 mg/L are the most effective treatments for improving nutritional composition^64^. According to Nofal et al.^63^ the order of the effects of different zinc sources on the zinc content of plant shoots is ZnO-NP > Zn-EDTA > ZnSO_4_. Also, the highest Zn levels were seen in leaves treated with 30 mg/L of MnO_2_-NPs^14^.

### Nutrient content in grains

The present investigation found that Nano-Mo 40 mg/l enhanced the protein and nitrogen content of maize grains, which is related to the role of molybdenum in protein synthesis and enzyme composition. According to El-Khouly^65^, the protein responded significantly to the NFs form of the administered nutrition when compared to the untreated control. The findings of the study also demonstrated that the majority of treatments, such as traditional and nano fertilization, increased the amount of phosphorus and potassium in maize grains. When NFs were treated at 30 mg/L^48^. peanut seeds had the highest levels of N, P, and K. Additionally, seed phosphorus absorption was markedly enhanced by the application of 500 or 1000 mg/L nano K and 75 mg/L nano Fe^65^.

The nano form of Zinc or manganese foliar sprays improves the absorption of iron and copper in maize grains. However, whether using nano or conventional fertilizer, the zinc and manganese content of maize grains increased more with foliar spraying Nano-Zn 40 mg/L, Zn-chelate 2 g/L, Nano-Zn 20 mg/L, Nano-Mn 40 mg/L, Nano-Mn 20 mg/L, and Mn-chelate 2 g/L. Plants require zinc, which is also involved in the creation of proteins and the function of many different enzymes. The length and biomass of plants are increased by 100 mg/L of ZnO-NPs, according to Srivastav et al.^66^. Dimkpa et al.^67^, found that nano-Mn foliar treatment greatly increased grain Mn transport efficiency, which may improve plant response control. Applying NFs at 40 mg/L produced the highest level of Fe, Mn, and Zn^48^. NPs can be tailored to increase plants’ access to and absorption of vital nutrients^19^.

### Nutrient content in soil

This study showed that the treatments with Nano-Mn and Mo enhanced the content of nitrogen in calcareous soil. Additionally, treatments with Nano-Mo and Zn increased the amount of phosphorus while Nano-Mo showed the greatest increase in accessible potassium in calcareous soil. Crop yields are increased by nanoparticles because they improve plant uptake, minimize environmental losses, and make it easier to distribute nutrients and growth regulators^68^. The soil’s iron content improved with the addition of Nano-Zn 40 mg/L. The content of micronutrients in the soil, such as zinc, manganese, or molybdenum, was preferred in treatments containing the same nutrient, whether in nano or traditional form. Zinc adsorption on calcareous soil is a great problem. Zn-NFs facilitate intelligent supply to plants and reduce zinc fixation in soil. Additionally, manganese in nanoform enhances its ability to pass through plant cell pores, while molybdenum boosts enzymatic responsiveness and nitrogen assimilation. The study found that increased positive absorption areas promote nutrient absorption and internal balance, consistent with previous research^67^ the application of nano Mn in foliar form significantly impacted the Mn level. It has been discovered that NPs improve absorption efficiency and direct active compounds to particular organelles and cell compartments in plants^69^. The NFs were characterized by low surface-mass ratio, which improves the absorption of nutrients by roots^70^. According to Srivastav, et al.^66^, ZnO NPs (100 mg/L) may encourage plant growth and be suggested as a zinc fertilizer source for agricultural production.

## Conclusions

Nano-micronutrient fertilizers (Nano-Zn, Mn, and Mo) with high doses enhanced vegetative growth and cob characteristics, as well as the yield components of the maize crop grown on calcareous soil. Furthermore, NFs improved the nutrient content in the leaves and the grains of the maize plant, in addition to the soil nutrients. In response to environmental cues, NFs use targeted, regulated, or slow-release mechanisms to increase the efficiency of nutrient utilization. They have positive effects at low concentrations and have a slim probability of bioaccumulating in the soil. As a result, more studies should be done on NFs as an alternative to chemical fertilizers, to encourage a more ecologically friendly way of farming. Finally, because of their advantages for the environment, ease of use, quick plant penetration, soil availability, and cost savings, NFs are suggested to be used as a substitute for conventional fertilizers.

## Data Availability

All data generated or analyzed during this study are included in this published article.
